# Risk Factor Analysis Based on Disease Severity: Rosacea Disease Management Strategies and Personalized Recommendations

**DOI:** 10.1111/jocd.70525

**Published:** 2025-12-24

**Authors:** Hongshan Liu, Luyue Zhang, Jianing Yuan, Jingchen Liang, Yuxin Zhang, Ziyun Gao, Ying Chen, Yawen Wang, Youbao Li, Weihui Zeng, Fan Yang

**Affiliations:** ^1^ Department of Dermatology The Second Affiliated Hospital of Xi'an Jiaotong University Xi'an China; ^2^ Department of Internal Medicine The First Affiliated Hospital of Xi'an Jiaotong University Xi'an China; ^3^ Department of Dermatology Shaanxi Provincial People's Hospital Xi'an China

**Keywords:** CEA, GFSS, IGA, PSA, risk factors, severity of rosacea

## Abstract

**Background:**

Rosacea is a common chronic inflammatory skin disease with complex causes and varied clinical manifestations. While risk factors for its onset have been studied, key factors associated with disease severity remain unclear. Identifying these factors is essential for optimizing management and treatment strategies.

**Aims:**

This study aims to identify the key risk factors affecting rosacea severity, characterize the clinical features of rosacea patients, and analyze the correlation between skin parameters and disease severity using the DermaLab system. The findings will provide a scientific basis for improving rosacea management.

**Methods:**

A cross‐sectional analysis was conducted on 305 newly diagnosed rosacea patients, collecting demographic data, lifestyle habits, and family history. Ordered logistic regression was used to identify key factors influencing disease severity, while the DermaLab system measured skin parameters and verified their correlation with disease severity.

**Results:**

The study found that gender, family history, hyperlipidemia, constipation, and allergic symptoms were significantly associated with rosacea severity. Regression analysis identified 10 key risk factors, with further verification confirming nine core factors. Additionally, certain dermatological parameters were positively correlated with disease severity, offering new insights for objective disease assessment.

**Conclusions:**

Rosacea management should focus on identifying risk factors through targeted strategies, including skincare and managing comorbidities. Personalized treatment plans can help control disease progression and improve patients' quality of life.

## 1 Introduction

Rosacea is a chronic inflammatory skin disease that primarily affects the central areas of the face, such as the cheeks, chin, nose, and forehead, as well as the eyes [1]. A recent study indicated that the global overall prevalence of rosacea is around 5.1% [2], mainly affecting women between 45 and 60 years old [3]. Its skin manifestations include temporary or persistent facial erythema, telangiectasia, edema, papules, and pustules. According to the definition by the Expert Committee on Classification and Staging of Rosacea of the National Rosacea Society, rosacea can be classified into four subtypes: Erythematotelangiectatic Rosacea (ETR), Papulopustular Rosacea (PPR), Phymatous Rosacea (PHY), and Ocular Rosacea [4]. Clinical presentations vary widely, and some patients may exhibit multiple subtypes concurrently. While ETR and PPR are relatively more common, ocular rosacea and phymatous changes deserve particular attention. In certain populations, the prevalence of rosacea reaches 22%, while ocular involvement may affect 50% or more [5]. Ocular rosacea manifests as inflammation of the ocular surface, including blepharitis and tear film instability, leading to symptoms such as irritation, redness, dryness, and conjunctivitis [6]. Severe complications, such as corneal ulcers, can impair quality of life and threaten vision [7, 8]. Phyma, often regarded as the advanced stage of rosacea, is more common in men [3], potentially mediated by increased androgen activity. Within phyma, rhinophyma is the most typical presentation, characterized by hypertrophy of nasal soft tissue, erythema, telangiectasia, nodules, and lobulation, producing a bulbous appearance; in severe cases, collapse of the external nasal valve may lead to nasal obstruction [9].

Currently, the pathogenesis of rosacea is not fully understood, but studies have confirmed its complex interactions with genetic factors, immune dysregulation, neurovascular dysregulation, microbial influences, and environmental factors [10]. Activation of immune‐mediated inflammatory pathways plays a central role, with recent studies highlighting the importance of mast cells (MCs) in disease pathophysiology [11]. Specifically, MRGPRX2 has been identified as a receptor for LL37, which can induce MC degranulation and promote the release and chemotaxis of multiple inflammatory mediators [12]. In addition, accumulating evidence indicates that the gut microbiota contributes significantly to rosacea pathophysiology [13]. Metabolic pathways are also implicated, as activation of the mTORC1 pathway—a proposed mechanism of rosacea—may be linked to adiponectin deficiency, leading to elevated S6 phosphorylation in the epidermis [14]. Additionally, genetic susceptibility and external factors such as ultraviolet exposure, high temperatures, and diet [15] may also contribute to the worsening of the condition.

In recent years, rosacea has garnered widespread attention due to its tendency to recur and its difficulty to treat. However, we find that current research on the risk factors for rosacea tends to focus on single factors or meta‐analyses. Numerous studies have explored the relationship between rosacea onset and factors such as smoking, alcohol consumption, specific medications, caffeine intake, and tea consumption. Some researchers have also conducted studies on specific populations, such as a study in China that limited its subjects to government employees to investigate the association with rosacea [16]. However, existing research on rosacea has mainly focused on its presence or absence, and studies incorporating multifactorial analysis remain relatively limited. As rosacea is difficult to manage and prone to progression, attention to factors associated with its severity is particularly important. This study explores the relationship between rosacea severity and relevant risk factors, offering a more comprehensive understanding of the condition. By identifying potential predictors, it supports clinicians in tailoring treatment strategies and helps patients better manage their condition through skincare, lifestyle adjustments, and awareness of comorbidities. These findings provide useful guidance for clinical practice and long‐term management of rosacea.

## 2 Materials and Methods

### 2.1 Study Design and Participants

**FIGURE 1:**
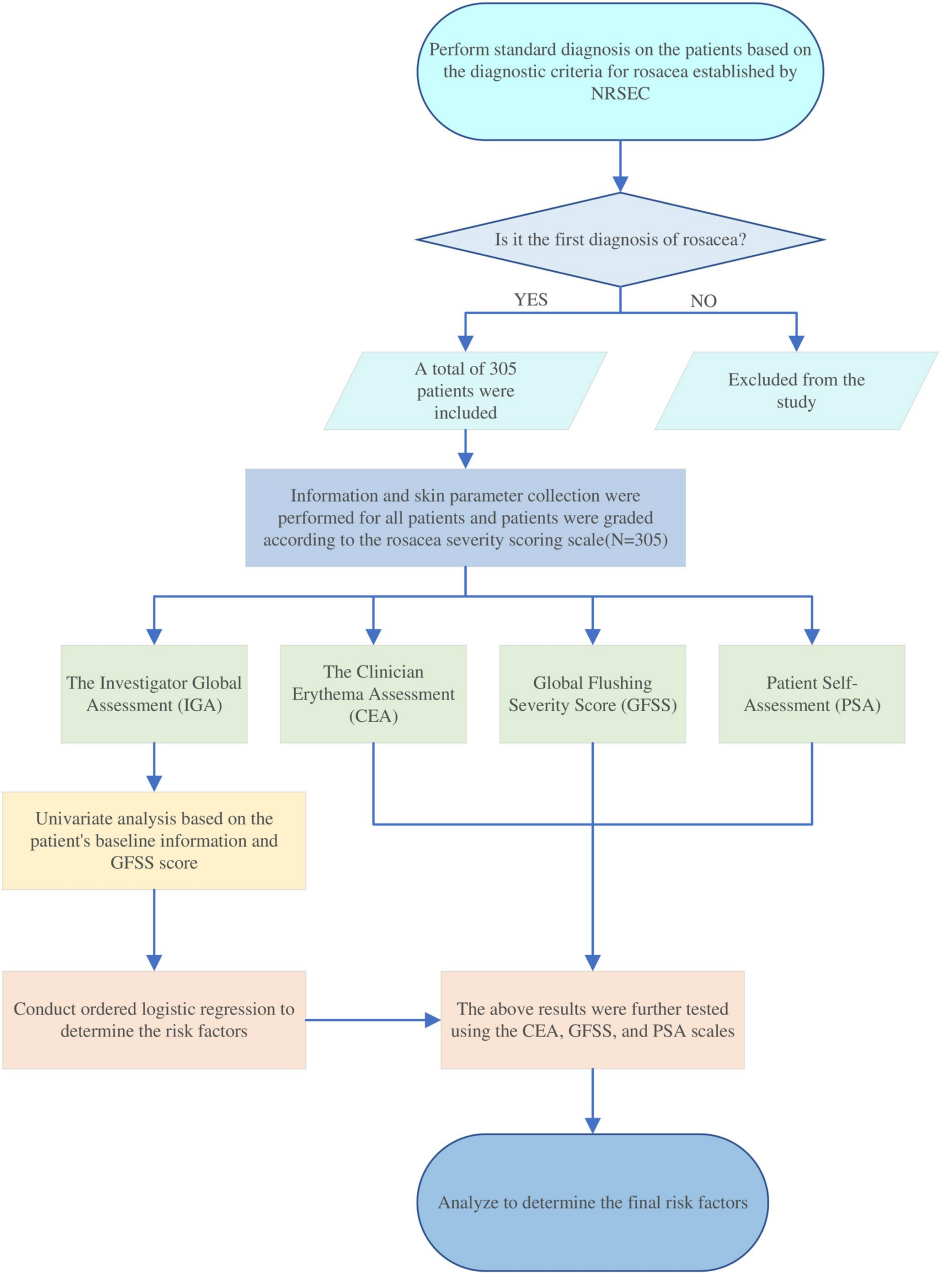
Flowchart of the study design.

In this cross‐sectional study, we included 305 newly diagnosed patients who met the diagnostic criteria for rosacea. The exclusion criteria mainly involved patients with other concomitant facial skin diseases and mental health disorders. The study flowchart is shown below (Figure 1). Baseline data were collected through interviews, including demographics, comorbid conditions, lifestyle, and psychological status, while dermatologists assessed the skin condition and clinical features. Informed consent was obtained from all participants, with each patient signing the consent form. The study was approved by the Ethics Committee of the Second Affiliated Hospital of Xi'an Jiaotong University (Ethics Approval No: 2023055), in accordance with the Declaration of Helsinki.

### 2.2 Diagnosis of Rosacea

The clinical diagnosis of rosacea was made by dermatologists at our hospital based on the patient's clinical symptoms, medical history, family history, along with corresponding auxiliary tests (such as dermoscopy and mite [Demodex]) and physical assessments. To ensure the accuracy of the study, all patients were newly diagnosed cases. The diagnosis and classification of rosacea followed the standards set by the National Rosacea Society Expert Committee (NRSEC) [4].

### 2.3 Data Collection

In the interview, data on patients' age, gender, family history, body mass index (BMI), annual personal income, dietary habits, drinking status, psychological status, daily facial care routine, skin condition, and comorbidities were collected. Age was categorized into < 24 years, 25–34 years, 35–44 years, and ≥ 45 years; BMI was calculated as weight (kg) divided by the square of height (m), and categorized as underweight (BMI < 18.5), normal (18.5 ≤ BMI < 24), and overweight or obese (BMI ≥ 24) [17]; personal annual income (X) was divided into four categories: X < 50 000, 50 000 ≤ X < 100 000, 100 000 ≤ X < 200 000, and X > 200 000; dietary habits included preferences for high‐fat foods and dairy products; psychological status was assessed using the DASS‐21 (depression, anxiety, and stress scale), with scores measuring the severity of anxiety, depression, and stress [18, 19]. Hypertension and hyperlipidemia were determined based on the patient's previous examination results, constipation was diagnosed as fewer than three bowel movements per week, and sleep problems were defined as the presence or absence of common sleep‐related issues, including insomnia, circadian rhythm disturbances, sleep‐disordered breathing, hypersomnia/narcolepsy, parasomnias, restless legs syndrome/periodic limb movement disorder and so on [20]. Skin condition was assessed by whether the patient had experienced allergic symptoms (such as erythema, wheals, pruritus, or other urticaria‐like symptoms) or sunburns in the past year. All of the above were self‐reported by the patients. Daily skincare habits included the use of acid products and face masks, water temperature, and cleaning frequency. We referenced the 2017 ROSCO Group's recommendations on the diagnosis, classification, and assessment of rosacea [21] and the updated standard management options for rosacea by the NRSEC in 2019 [22]. The collected clinical phenotypic characteristics included transient flushing, persistent erythema, papules/pustules, phymatous changes, telangiectasia, and subjective symptoms such as pain, itching, and burning sensation. Due to the complexity of the severity scoring system for rosacea, this study will incorporate multiple mainstream scales to assess and analyze the severity of rosacea, including the Investigator Global Assessment (IGA) scale [23], the Clinician Erythema Assessment (CEA) score [24], Global Flushing Severity Score (GFSS) [25], and Patient Self‐Assessment (PSA) scales [26], which were used as a patient‐reported outcome measure. Among these, the IGA score, as a widely recognized tool for assessing overall disease severity, will serve as the primary scale for analysis, while the other scales will be used for further validation of the results. To ensure the authenticity and validity of the collected data, at least one dermatologist will accompany the interviews throughout, providing guidance and completing the assessments. In addition, the DermaLab Combo3 system (Cortex Technology, Hadsund, Denmark) was used to assess the EI, TEWL, and hydration index (HI). For each patient, measurements were taken at four standardized sites on both the left and right sides of the face: the forehead, cheek, nasal ala, and chin.

### 2.4 Statistical Analysis

Statistical analysis was performed using SPSS 26 (IBM SPSS, USA). Measurement data were expressed as means and standard deviations, while count data were expressed as frequencies and percentages. The rank‐sum test was used for univariate analysis. Using the severity of rosacea as the dependent variable, statistically significant results from the univariate analysis were incorporated into an ordered logistic regression to calculate the adjusted odds ratios (aOR) and 95% confidence intervals (CI). The correlation between skin physiology parameters and disease severity was assessed using Spearman's correlation analysis. A correlation of at least 0.70 was considered very strong, 0.40–0.69 as strong, 0.30–0.39 as moderate, 0.20–0.29 as weak, and 0.01–0.19 as no or negligible correlation. A *p*‐value < 0.05 was considered statistically significant.

## 3 Results

This study included a total of 305 newly diagnosed rosacea patients. Based on the primary assessment scale, the IGA score results showed the following distribution of disease severity: no patients scored 0, 25 patients were rated 1 (8.2%), 124 patients were rated 2 (40.7%), 108 patients were rated 3 (35.4%), and 48 patients were rated 4 (15.7%). The CEA scale results showed that 7 patients scored 0 (2.3%), 47 patients scored 1 (15.4%), 119 patients scored 2 (39.0%), 99 patients scored 3 (32.5%), and 33 patients scored 4 (10.8%). The GFSS scale results showed that 20 patients scored 0 (6.6%), 102 patients scored 1 (33.4%), 129 patients scored 2 (42.3%), and 54 patients scored 3 (17.7%). Additionally, PSA scale scores were also collected. Finally, the study performed a comprehensive analysis of the demographic data and clinical information.

### 3.1 Demographic Characteristics and Clinical Features

**FIGURE 2:**
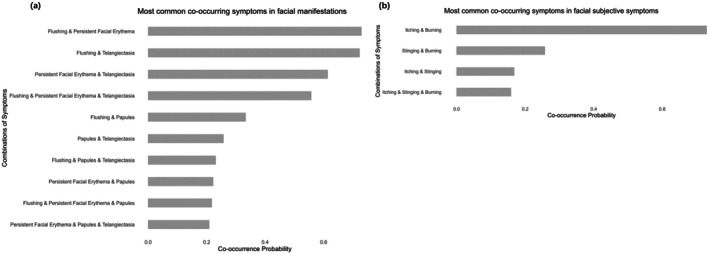
The co‐occurrence probability of most commonly observed clinical symptoms. (a) Shows the combinations with a higher co‐occurrence probability among facial signs. (b) Shows the combinations with a higher co‐occurrence probability among subjective.

This study included 305 rosacea patients, with a male‐to‐female ratio of 0.24 (see Table 1 for details). The proportion of patients with facial flushing and persistent erythema was 92.13% and 80.66%, respectively; telangiectasia occurred in 78.36%, while papules and pustules occurred in 34.43% and 18.69%, respectively. Most patients had lesions on the cheeks (92.79%) and nose (81.31%). The most commonly reported skin symptoms were burning (94.75%), itching (78.03%), and stinging (26.89%). In addition, we further divided the patients based on their clinical symptoms into two groups: the clinical facial signs group, including flushing, persistent facial erythema, telangiectasia, papules, pustules, and phymatous changes; and the clinical subjective symptoms group, including burning, itching, and stinging. We also analyzed the co‐occurrence probability of different symptoms within each group. Figure 2a,b presents the symptom combinations with higher co‐occurrence probabilities. It can be seen that the co‐occurrence of flushing and persistent facial erythema is relatively high among clinical signs, while itching and burning sensations are the most commonly associated subjective symptoms in patients.

**TABLE 1:** Demographic characteristics of subjects.

Variables	Number of subjects	*N* (%)
Gender, *n* (%)		
Male	60	19.7
Female	245	80.3
Age (years), *n* (%)		
< 24	27	8.9
25–34	152	49.8
35–44	65	21.3
> 44	61	20
BMI (kg/m^2^), *n* (%)		
< 18.5	49	16.1
18.5–24	214	70.2
≥ 24	42	13.8
With family history or not, *n* (%)		
Yes	38	12.5
No	267	87.5
Annual income (10 000/per year), *n* (%)		
< 5	79	25.9
5–10	104	34.1
10–20	84	27.5
> 20	38	12.5
With hypertension or not, *n* (%)		
Yes	34	11.1
No	271	88.9
With hyperlipidemia or not, *n* (%)		
Yes	108	35.4
No	197	64.6
With constipation or not, *n* (%)		
Yes	71	23.3
No	234	76.7
With allergic symptoms or not, *n* (%)		
Yes	154	50.5
No	151	49.5
Presence of sleep problems, *n* (%)		
Yes	140	45.9
No	165	54.1
Preference for high‐fat foods or not, *n* (%)		
Yes	131	43.0
No	174	57.0
Preference for dairy products or not, *n* (%)		
Yes	161	52.8
No	144	47.2
Drinking status, *n* (%)		
Yes	78	25.6
No	227	74.4
Use acidic skincare products or not, *n* (%)		
Yes	92	30.2
No	213	69.8
Face washing frequency (/per day), *n* (%)		
≤ 1	95	31.1
=2	200	65.6
> 2	10	3.3
Water temperature when cleaning, *n* (%)		
Cold water	78	25.6
Warm water	198	64.9
Hot water	29	9.5
Adhere to applying facial mask or not, *n* (%)		
Yes	123	40.3
No	182	59.7
Sunburn condition, *n* (%)		
Yes	190	62.3
No	115	37.7
The degree of anxiety, *n* (%)		
Normal	181	59.3
Mild anxiety	23	7.5
Moderate anxiety	56	18.4
Severe anxiety	16	5.2
Extremely anxiety	29	9.5
The degree of anxiety, *n* (%)		
Normal	181	59.3
Mild anxiety	23	7.5
Moderate anxiety	56	18.4
Severe anxiety	16	5.2
Extremely anxiety	29	9.5
The degree of anxiety, *n* (%)		
Normal	181	59.3
Mild anxiety	23	7.5
Moderate anxiety	56	18.4
Severe anxiety	16	5.2
Extremely anxiety	29	9.5
The degree of pressure, *n* (%)		
Normal	244	80.0
Mild pressure	18	5.9
Moderate pressure	19	6.2
Severe pressure	16	5.2
Extremely pressure	8	2.6
The degree of depression, *n* (%)		
Normal	231	75.7
Mild depression	36	11.8
Moderate depression	26	8.5
Severe depression	4	1.3
Extremely depression	8	2.6

Abbreviation: BMI, body mass index.

### 3.2 The Correlation Between TEWL, HI, EI, MI and Disease Severity

The overall final mean ± standard deviation for each parameter was as follows: TEWL = 22.79 ± 5.24, EI = 17.07 ± 1.46, MI=20.19 ± 3.97 and HI = 146.30 ± 19.06. Spearman correlation analysis revealed a moderate positive correlation between TEWL and the severity of rosacea (*r* = 0.385), indicating that higher TEWL values may correspond to more severe disease. A moderate negative correlation was found between HI and rosacea severity (*r* = −0.301), suggesting that higher hydration levels may be associated with less severe disease. A strong correlation was observed between EI and rosacea severity (*r* = 0.735), while no significant correlation was found between MI and rosacea severity (Table 2).

**TABLE 2:** Spearman correlation analysis between TEWL, HI, EI, MI, and disease severity.

	Correlation coefficient		*N*	*p*
	Variables	IGA Scores		
Spearman correlation	IGA Scores	1.000		
	TEWL	0.385	305	< 0.001
	EI	0.735	305	< 0.001
	MI	0.043	305	0.458
	Hydration	−0.301	305	< 0.001

Abbreviations: EI, erythema index; IGA, Investigator Global Assessment; MI, melanin index; TEWL, transepidermal water los.

### 3.3 Ten Risk Factors Associated With the Severity of Rosacea Were Identified Through the IGA Scale Analysis

Our univariate analysis included 21 risk factors, analyzed using the rank sum test with IGA score as the outcome variable. The results showed significant differences in 14 factors: gender (*p* = 0.003), annual income (*p* = 0.03), family history (*p* = 0.001), history of hyperlipidemia (*p* = 0.001), preference for high‐fat foods (*p* = 0.034), preference for dairy products (*p* = 0.036), history of sleep disorders (*p* = 0.037), alcohol consumption (*p* = 0.04), history of constipation (*p* < 0.001), use of acidic products (*p* = 0.001), allergic conditions (*p* < 0.001), frequency of face washing (*p* < 0.001), water temperature during face washing (*p* < 0.001), and stress levels (*p* < 0.001) (Table 3).

**TABLE 3:** Univariate analysis of risk factors related to the severity of rosacea.

Variables	IGA score					
	1 (*N* = 25)	2 (*N* = 124)	3 (*N* = 108)	4 (*N* = 48)	*H*	*p*
Gender						
Male	10 (40.0)	27 (21.8)	19 (17.6)	4 (8.3)	−2.945	0.003
Female	15 (60.0)	97 (78.2)	89 (82.4)	44 (91.7)		
Age (years)						
< 24	0 (0.0)	11 (8.9)	12 (11.1)	4 (8.3)	1.336	0.721
25–34	14 (56.0)	60 (48.4)	51 (47.2)	27 (56.3)		
35–44	5 (20.0)	26 (21.0)	27 (25.0)	7 (14.6)		
> 44	6 (24.0)	27 (21.8)	18 (16.7)	10 (20.8)		
BMI (kg/m^2^)						
< 18.5	6 (24.0)	14 (11.3)	18 (16.7)	11 (22.9)	4.287	0.117
18.5–24	17 (68.0)	95 (76.6)	75 (69.4)	27 (56.3)		
≥ 24	2 (8.0)	15 (12.1)	15 (13.9)	10 (20.8)		
With family history or not						
Yes	4 (16.0)	6 (4.8)	13 (12.0)	15 (31.3)	−3.411	0.001
No	21 (84.0)	118 (95.2)	95 (88.0)	33 (68.8)		
Annual income (10 000/per year)						
< 5	7 (28.0)	27 (21.8)	28 (25.9)	17 (35.4)	8.982	0.03
5–10	4 (16.0)	40 (32.3)	44 (40.7)	16 (33.3)		
10–20	8 (32.0)	38 (30.6)	27 (25.0)	11 (22.9)		
> 20	6 (24.0)	19 (15.3)	9 (8.3)	4 (8.3)		
With hypertension or not						
Yes	2 (8.0)	16 (12.9)	8 (7.4)	8 (16.7)	−0.228	0.819
No	23 (92.0)	108 (87.1)	100 (92.6)	40 (83.3)		
With hyperlipidemia or not						
Yes	11 (44.0)	2 (20.2)	45 (41.7)	27 (56.3)	−3.703	< 0.001
No	14 (56.0)	99 (79.8)	63 (58.3)	21 (43.8)		
With constipation or not						
Yes	5 (20.0)	20 (16.1)	24 (22.2)	22 (45.8)	−3.316	0.001
No	20 (80.0)	104 (83.9)	84 (77.8)	26 (54.2)		
With allergic symptoms or not						
Yes	10 (40.0)	49 (39.5)	60 (55.6)	35 (72.9)	−4.030	< 0.001
No	15 (60.0)	75 (60.5)	48 (44.4)	13 (27.1)		
Presence of sleep problems						
Yes	12 (48.0)	65 (52.4)	47 (43.5)	16 (33.3)	−2.090	0.037
No	13 (52.0)	59 (47.6)	61 (56.5)	32 (66.7)		
Preference for high‐fat foods or not						
Yes	11 (44.0)	44 (35.5)	49 (45.4)	27 (56.3)	−2.123	0.034
No	14 (56.0)	80 (64.5)	59 (54.6)	21 (43.8)		
Preference for dairy products or not						
Yes	10 (40.0)	50 (40.3)	59 (54.6)	25 (52.1)	−2.098	0.036
No	15 (60.0)	74 (59.7)	49 (45.4)	23 (47.9)		
Drinking status						
Yes	6 (24.0)	25 (20.2)	29 (26.9)	18 (37.5)		
No	19 (76.0)	99 (79.8)	79 (73.1)	30 (62.5)	−2.058	0.040
Use acidic skincare products or not						
Yes	14 (56.0)	41 (33.1)	31 (28.7)	6 (12.5)	−3.474	0.001
No	11 (44.0)	83 (66.9)	77 (71.3)	42 (87.5)		
Face washing frequency (/per day)						
≤ 1	5 (20.0)	29 (23.4)	40 (37.0)	21 (43.8)	25.133	< 0.001
=2	20 (80.0)	95 (76.6)	63 (58.3)	22 (45.8)		
> 2	0 (0.0)	0 (0.0)	5 (4.6)	5 (10.4)		
Water temperature when cleaning						
Cold water	5 (20.0)	22 (17.1)	31 (28.7)	20 (41.7)	29.463	< 0.001
Warm water	20 (80.0)	97 (78.2)	62 (57.4)	19 (39.6)		
Hot water	0 (0.0)	5 (4.0)	15 (13.9)	9 (18.8)		
Adhere to applying facial mask or not						
Yes	14 (56.0)	46 (37.1)	42 (38.9)	21 (43.8)	−0.216	0.829
No	11 (44.0)	78 (62.9)	66 (161.1)	27 (56.3)		
Sunburn condition						
Yes	15 (60.0)	82 (66.1)	69 (63.9)	24 (50.0)	−1.286	0.198
No	10 (40.0)	42 (33.9)	39 (36.1)	24 (50.0)		
The degree of anxiety						
Normal	16 (64.0)	76 (61.3)	63 (58.3)	26 (54.2)	1.860	0.761
Mild anxiety	2 (8.0)	7 (5.6)	9 (8.3)	5 (10.4)		
Moderate anxiety	5 (20.0)	23 (18.5)	16 (14.8)	12 (25.0)		
Severe anxiety	1 (4.0)	7 (5.6)	7 (6.5)	1 (2.1)		
Extremely anxiety	1 (4.0)	11 (8.9)	13 (12.0)	4 (8.3)		
The degree of pressure						
Normal	23 (92.0)	108 (87.1)	81 (75.0)	32 (66.7)	20.497	< 0.001
Mild pressure	1 (4.0)	7 (5.6)	7 (6.5)	3 (6.3)		
Moderate pressure	1 (4.0)	5 (4.0)	10 (9.3)	3 (6.3)		
Severe pressure	0 (0.0)	4 (3.2)	7 (6.5)	5 (10.4)		
Extremely pressure	0 (0.0)	0 (0.0)	3 (2.8)	5 (10.4)		
The degree of depression						
Normal	24 (96.0)	91 (73.4)	82 (75.9)	34 (70.8)	2.339	0.674
Mild depression	0 (0.0)	18 (14.5)	11 (10.2)	7 (14.6)		
Moderate depression	1 (4.0)	11 (8.9)	9 (8.3)	5 (10.4)		
Severe depression	0 (0.0)	1 (0.8)	2 (1.9)	1 (2.1)		
Extremely depression	0 (0.0)	3 (2.4)	4 (3.7)	1 (2.1)		

*Note:* Statistically significant at *p* < 0.05, analysis of rank‐sum test.

Abbreviation: BMI, body mass index.

**FIGURE 3:**
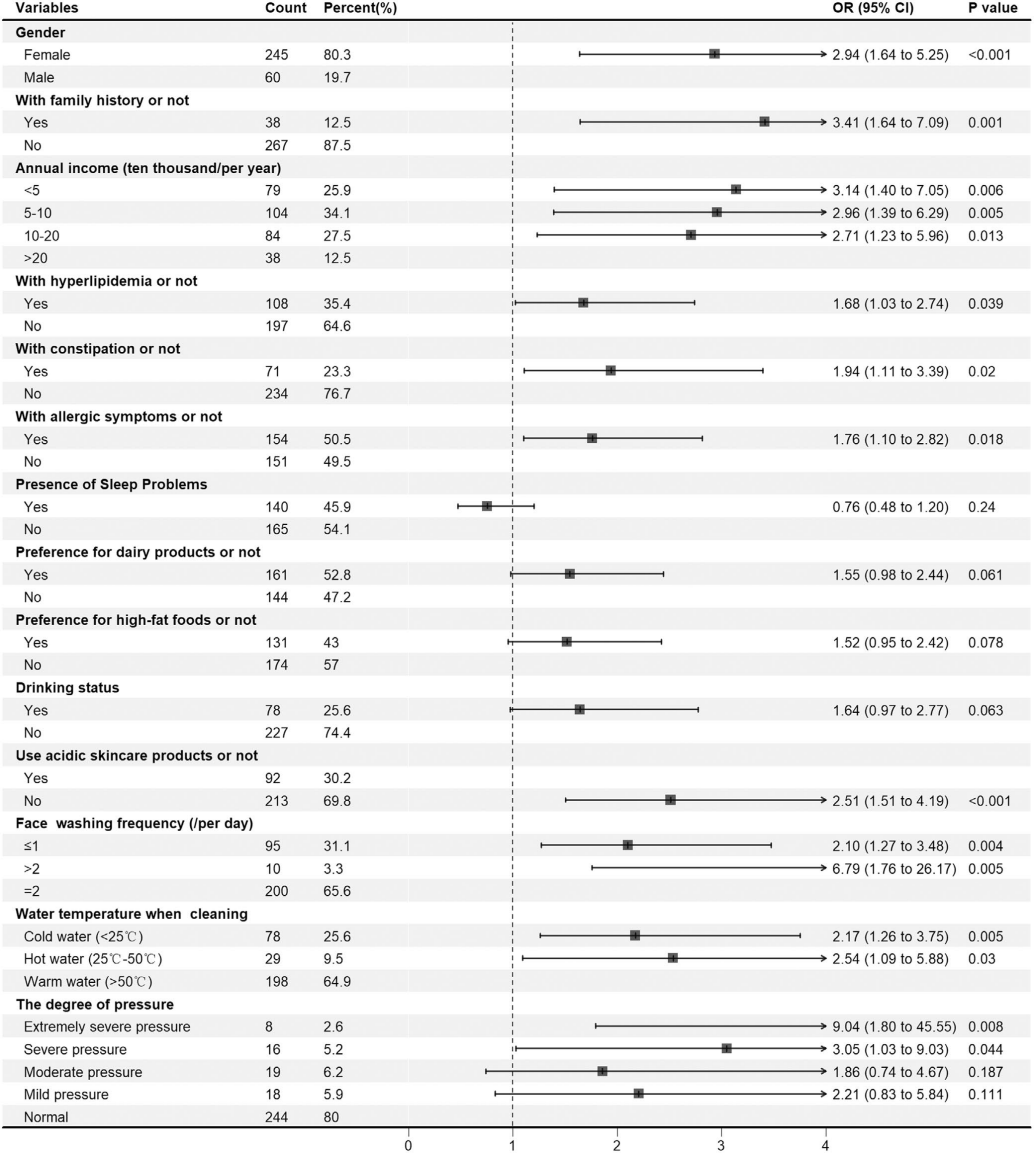
Forest plot of risk factors derived from multivariate regression analysis based on the IGA scale. Estimates are odds ratio (OR) and 95% CI. Statistically significant at *p* < 0.05, analysis of ordered logistic regression.

Multivariate analysis included 14 statistically significant factors from the univariate analysis. The results identified the following 10 potential risk factors related to disease severity: female gender (*p* < 0.001), family history (*p* = 0.001), low annual income, history of hyperlipidemia (*p* = 0.038), history of constipation (*p* = 0.02), never using acidic products (*p* < 0.001), allergic comorbidities (*p* = 0.018), incorrect face‐washing frequency, inappropriate water temperature, and high stress levels (Figure 3).

### 3.4 Nine Risk Factors Were Ultimately Identified Through the Combination of the CEA Scale, GFSS Scale, and PSA Scale

The subsequent evaluation analysis based on the CEA, GFSS, and PSA scales showed that, except for low income levels, nine other risk factors remained statistically significant (*p* < 0.05) (Table 4). Therefore, they were identified as key risk factors closely related to the severity of rosacea.

**TABLE 4:** Multivariate regression analysis of risk factors based on CEA, GFSS, and PSA scales.

	CEA		GFSS		PSA	
	OR (95% CI)	*p*	OR (95% CI)	*p*	OR (95% CI)	*p*
Gender						
Male	Ref		Ref		Ref	
Female	1.305 (1.035, 1.645)	0.024	1.280 (1.053, 1.566)	0.013	1.882 (1.088, 3.176)	0.024
With family history or not						
Yes	1.456 (1.099, 1.930)	0.009	1.367 (1.078, 1.734)	0.010	2.202 (1.097, 4.421)	0.026
No	Ref		Ref		Ref	
Annual income (10 000/per year)						
< 5	1.049 (0.761, 1.446)	0.771	1.182 (0.902, 1.549)	0.226	1.634 (0.766, 3.485)	0.204
5–10	1.261 (0.930, 1.709)	0.135	1.147 (0.888, 1.482)	0.295	1.940 (0.950, 3.963)	0.069
10–20	1.149 (0.836, 1.580)	0.393	1.181 (0.903, 1.545)	0.225	1.237 (0.592, 2.586)	0.572
> 20	Ref		Ref		Ref	
With hyperlipidemia or not						
Yes	1.233 (1.010, 1.504)	0.039	1.265 (1.070, 1.496)	0.006	1.734 (1.073, 2.801)	0.025
No	Ref		Ref		Ref	
With constipation or not						
Yes	1.382 (1.105, 1.728)	0.005	1.222 (1.012, 1.475)	0.037	1.832 (1.1056, 3.176)	0.031
No	Ref		Ref		Ref	
Use acidic skincare products or not						
Yes	Ref		Ref		Ref	
No	1.309 (1.070, 1.601)	0.009	1.457 (1.229, 1.727)	< 0.001	2.121 (1.297, 3.468)	0.003
With allergic symptoms or not						
Yes	1.247 (1.031, 1.508)	0.023	1.201 (1.024, 1.410)	0.025	1.616 (1.029, 2.537)	0.037
No	Ref		Ref		Ref	
Face washing frequency (/per day)						
≤ 1	1.356 (1.108, 1.660)	0.003	1.266 (11.068, 1.501)	0.007	1.878 (1.160, 3.040)	0.010
> 2	1.802 (1.058, 3.069)	0.030	1.902 (1.214, 2.980)	0.005	3.581 (1.069, 12.001)	0.039
=2	Ref		Ref		Ref	
Water temperature when cleaning						
Cold water	1.266 (1.109, 1.574)	0.033	1.491 (1.241, 1.791)	< 0.001	1.845 (1.101, 3.094)	0.020
Hot water	1.513 (1.068, 2.144)	0.020	1.413 (1.053, 1.895)	0.021	2.478 (1.074, 5.717)	0.033
Warm water	Ref		Ref		Ref	
The degree of pressure						
Normal	Ref		Ref		Ref	
Mild pressure	0.850 (0.572, 1.264)	0.422	1.046 (0.784, 1.461)	0.794	2.594 (1.050, 6.408)	0.039
Moderate pressure	1.206 (0.821, 1.773)	0.339	1.203 (0.870, 1.664)	0.264	1.615 (0.664, 3.926)	0.290
Severe pressure	1.545 (1.012, 2.359)	0.044	1.641 (1.149, 2.345)	0.007	5.736 (1.940, 16.963)	0.002
Extremely pressure	1.920 (1.053, 3.503)	0.033	1.818 (1.095, 3.018)	0.021	5.934 (1.420, 24.804)	0.015

*Note:* Statistically significant at *p* < 0.05, results of the ordered logistic analysis.

Abbreviations: BMI, body mass index; CI, confidence interval; OR, odds ratio.

## 4 Discussion

This study is a comprehensive analysis of the correlation between multiple risk factors and the severity of rosacea, providing practical recommendations for patient management. Previous studies have mostly focused on univariate analysis or research on specific populations and disease subtypes. For example, one study showed a negative correlation between relative muscle mass and rosacea severity [27], suggesting that skeletal muscle may have a protective effect against disease progression. Additionally, research has found that patients with rhinophyma have lower serum levels of GLA (gamma‐aminobutyric acid), which are negatively correlated with the severity of erythema [28]. In terms of phenotype analysis, one study explored the risk factors for nasal rosacea, showing that factors such as male gender, obesity, and alcohol consumption could be risk factors for this form of rosacea [17]. While these studies have provided valuable insights, our study, based on previous research, further enriches our understanding of rosacea by conducting multivariate analysis and considering multiple risk factors in relation to disease severity. It also provides new ideas for improving prognosis and enhancing patients' quality of life.

### 4.1 Attention to Condition Management and Control: A Focus for Female Patients and Those With a Family History

Rosacea is a common chronic inflammatory skin disease with a higher prevalence in women, particularly those of reproductive age [29, 30]. Epidemiological studies have shown that the risk of rosacea decreases significantly in postmenopausal women, suggesting a potential role of female sex hormones in disease pathogenesis [31]. Further experimental studies have demonstrated that estrogen supplementation can exacerbate rosacea‐related inflammatory responses. For instance, GPR30‐deficient mice exhibited reduced inflammation, while inhibition or knockdown of GPR30 significantly alleviated rosacea‐like inflammation in LL37 or LL37 plus E2‐treated models, indicating that activation of the E2/GPR30 signaling pathway plays a critical role in the inflammatory process [32]. In addition, pregnancy, a physiological state characterized by markedly elevated levels of female sex hormones such as estradiol, has been associated with an increased risk of severe manifestations of rosacea, including rosacea flares [33, 34]. Collectively, current evidence suggests that female sex may be correlated with both susceptibility to and severity of rosacea, which is consistent with the findings of our study.

Among the patients in our study, 12.5% had a family history of rosacea, and this proportion increased to 17.95% among those with moderate to severe conditions (GFSS scores of 2 and 3). This phenomenon suggests that family history may be associated with the severity of the condition. Some studies have indicated that family history is one of the important mechanisms for the onset of rosacea [35]. Furthermore, through whole‐genome sequencing, researchers identified three genes—LRRC4, SH3PXD2A, and SLC26A8—each of which has rare genetic variations associated with susceptibility to rosacea within families, indicating these genes as potential susceptibility genes [36]. This finding further clarifies the significant role of genetics in the development of rosacea. Patients with a family history face a higher potential risk compared to those without genetic backgrounds, suggesting that genetic factors have a relatively constant impact on the course of rosacea. Overall, the impact of gender and family history on the condition primarily occurs through the interaction of genetic and endocrine factors.

### 4.2 Focusing on Comorbidities: Managing Hyperlipidemia, Constipation, and Allergies in Rosacea Patients

Hyperlipidemia is characterized by elevated levels of lipids (fats) in the blood, including cholesterol and triglycerides, and is primarily associated with an increased risk of cardiovascular diseases [37]. In recent years, increasing evidence suggests a potential link between hyperlipidemia and skin diseases, with more research focusing on psoriasis. Studies indicate a possible positive correlation between hyperlipidemia and psoriasis [38, 39]. A case–control study also found that urticaria may be associated with a prior diagnosis of hyperlipidemia [40]. However, the relationship between hyperlipidemia and rosacea remains underexplored in the current literature. Vascular endothelial growth factor (VEGF) is believed to play a significant role in the development of atherosclerosis, with VEGF levels elevated in hyperlipidemic patients [41]. In rosacea, upregulated expression and secretion of VEGF promote neovascularization, exacerbating the symptoms of rosacea [42]. This may represent a potential mechanism linking hyperlipidemia with rosacea.

In addition, constipation is a common gastrointestinal issue characterized by difficulty in defecation, reduced bowel frequency, and hard stools [43], classified as a gastrointestinal dysfunction disorder. Studies have shown a significant association between gastrointestinal dysfunction (such as halitosis, gastroesophageal reflux, bloating, constipation) and sebaceous gland disorders [44], which may increase sebaceous secretion and promote the proliferation of lipophilic Malassezia [45], potentially contributing to the onset and progression of rosacea.

Rosacea is essentially a chronic acne‐like skin condition, where impaired skin barrier function leads to sensitivity to allergens. In this study, over half of the patients experienced varying degrees of allergic symptoms, such as itching or urticaria‐like changes, in the past 12 months. Additionally, rosacea symptoms in these patients were generally more severe. Studies have shown that rosacea patients have a higher prevalence of allergic contact dermatitis [46], suggesting a close relationship between rosacea and allergies.

In conclusion, the presence of these comorbidities may be related to the severity of rosacea. Therefore, effectively addressing and managing these conditions is necessary to improve the overall condition of rosacea patients.

### 4.3 Cleansing and Skincare Tips for Rosacea Patients: Pay Attention to Cleansing Frequency and Water Temperature, and Use Acidic Products in Moderation

Due to the sensitivity of rosacea patients' skin, which can react quickly to irritants, it is crucial to emphasize proper skincare methods in addition to standard patient education and pharmacological treatment [47]. Improper care and cleansing can exacerbate the condition of rosacea. First, rosacea patients should pay special attention to water temperature during daily cleansing; it should remain mild to avoid triggering vascular reactions [47]. Using lukewarm water for cleansing helps alleviate the symptoms of rosacea [48]. Compared to patients who cleanse with lukewarm water, those who use cold or hot water seem to have more severe symptoms, and this difference is statistically significant. This may relate to the dysregulation of the neurogenic, immune, and vascular mechanisms in rosacea patients.

Rosacea patients should pay special attention to the frequency of face washing. Although there is no specific study determining the optimal washing frequency, skincare experts generally recommend that rosacea patients wash their faces twice a day with a gentle cleanser, which helps alleviate the condition [48]. Another study explored the impact of cleansing frequency on male patients with mild to moderate acne. The results indicated that while there were no significant differences among the groups that cleansed once, twice, or four times daily, patients who cleansed twice a day showed significant improvement in acne lesions, while those who cleansed once a day experienced a worsening of their condition, supporting the recommendation for acne patients to cleanse their faces twice daily [49]. Similar findings were observed in the study of rosacea patients, where those who cleansed once a day or more frequently were more likely to experience exacerbation of their condition compared to those who cleansed twice a day, possibly due to skin barrier damage from improper or excessive cleansing [48]. Therefore, it is recommended that rosacea patients wash their faces twice a day to help control the condition.

In terms of skincare, the study found that 30.2% of patients regularly used salicylic acid products to improve skin conditions, and their symptoms appeared to be milder. Domestic studies have confirmed that supramolecular salicylic acid can improve inflammatory lesions caused by rosacea and aid in repairing the skin barrier [50]. A clinical study in China similarly indicated that supramolecular salicylic acid could improve the erythema index and overall skin appearance in rosacea patients [51], though most studies focus on PPR patients with more severe inflammation. Further research is needed on other rosacea subtypes.

### 4.4 Stress Management Tips for Rosacea Patients: Minimize Stress and Its Impact

Moreover, psychological factors have a close association with skin diseases. A previous study on government employees indicated that higher multiple social risk scores (PsRS) were associated with an increased risk of developing rosacea. Although there is no consistent conclusion regarding the association of low income on the severity of rosacea, some studies suggest that low income is associated with higher PsRS scores, which may lead to overcrowded living conditions and, in turn, affect mental health [16]. Currently, there is widespread acceptance in clinical practice of a close link between stress and rosacea [52]. Psychological stress may affect the homeostasis of the skin barrier, the integrity of the stratum corneum, and the innate immune function of the epidermis [52]. It can also act as an effective inflammatory activator, leading to increased cytokines, activating neurons and inflammatory pathways [52], this exacerbates inflammation and drives the progression of rosacea. At the same time, facial flushing may trigger social anxiety, and rosacea itself significantly impacts the patient's quality of life, leading to serious social and psychological burdens. Over time, psychological stress and disease progression may form a vicious cycle.

### 4.5 Study Limitations

This study has several limitations. First, as a hospital‐based, single‐center study, the patient sample may not fully represent the general population, which could lead to selection bias and limit the generalizability of the findings. Second, the study cohort consisted predominantly of female patients, with relatively fewer male participants, which may introduce gender‐related bias and affect the applicability of the results to both sexes. Third, some variables such as dietary habits, allergy history, and constipation were collected through self‐reported questionnaires, which may be subject to recall or reporting bias, thereby introducing potential information bias. Finally, due to its cross‐sectional design, the study can only demonstrate associations between variables and disease severity, but cannot establish causal relationships. Therefore, the conclusions should be interpreted with caution, and further validation through large‐scale, prospective, and multi‐center studies with objective measurement tools is warranted.

## 5 Conclusion

This study outlines the clinical characteristics of rosacea patients and identifies nine potential risk factors that may be associated with disease severity, including female gender, family history, hyperlipidemia, constipation, and allergic symptoms. Furthermore, the study found a significant positive correlation between TEWL, EI, and the severity of rosacea, while skin hydration levels showed a significant negative correlation. These findings are of great significance for the management and control of rosacea patients in the future.

## Author Contributions

Hongshan Liu and Luyue Zhang: Conceptualization. Jianing Yuan and Jingchen Liang: Methodology. Yuxin Zhang, Ziyun Gao and Youbao Li: Investigation. Hongshan Liu and Luyue Zhang: Writing – original draft. Ying Chen, Yawen Wang and Fan Yang: Writing – review and editing. Weihui Zeng: funding acquisition. Fan Yang: supervision.

## Funding

This work was supported by the Natural Science Basic Research Program of Shaanxi, 2023‐JC‐YB‐787; Shaanxi Administration of Traditional Chinese Medicine, SZY‐KJCYC‐2023‐062; Xi'an Science and Technology Plan Project, 2023JH‐YXYB‐0009.

## Ethics Statement

Ethics approval was obtained from the Ethics Committee of the Second Affiliated Hospital of Xi'an Jiaotong University (number: 2023055). All participants signed informed consent forms, and the study was conducted in accordance with the Declaration of Helsinki.

## Conflicts of Interest

The authors declare no conflicts of interest.

## Data Availability

Data sharing not applicable to this article as no datasets were generated or analyzed during the current study.
